# Fabrication and Characterization of a Highly-Sensitive Surface-Enhanced Raman Scattering Nanosensor for Detecting Glucose in Urine

**DOI:** 10.3390/nano8080629

**Published:** 2018-08-20

**Authors:** Yudong Lu, Ting Zhou, Ruiyun You, Yang Wu, Huiying Shen, Shangyuan Feng, Jingqian Su

**Affiliations:** 1 College of Chemistry and Materials Science, Fujian Key Laboratory of Polymer Materials, Fuzhou, Fujian 350007, China; zt1102801305@163.com (T.Z.); youruiyun@fjnu.edu.cn (R.Y.); 2 Center of Engineering Technology Research for Microalgae Germplasm Improvement of Fujian, Southern Institute of Oceanography, Fujian Normal University, Fuzhou, Fujian 350117, China; wuyang@fjnu.edu.cn (Y.W.); shenhy@fjnu.edu.cn (H.S.); 3 Fujian Key Laboratory of Innate Immune Biology, Biomedical Research Center of South China, College of Life Science, Fujian Normal University, Fuzhou, Fujian 350117, China; sjq027@fjnu.edu.cn; 4 Key Laboratory of Optoelectronic Science and Technology for Medicine of Ministry of Education, Fujian Provincial Key Laboratory of Photonics Technology, Fujian Normal University, Fuzhou, Fujian 350117, China; syfeng@fjnu.edu.cn (S.F.)

**Keywords:** Au@Ag core-shell nanostructure, 4-PATP, glucose, internal standard, surface-enhanced Raman scattering

## Abstract

Herein we utilized coordination interactions to prepare a novel core-shell plasmonic nanosensor for the detection of glucose. Specifically, Au nanoparticles (NPs) were strongly linked with Ag+ ions to form a sacrificial Ag shell by using 4-aminothiophenol (4-PATP) as a mediator, which served as an internal standard to decrease the influence of the surrounding on the detection. The resultant Au-PATP-Ag core-shell systems were characterized by UV-vis spectroscopy, transmission electron microscopy, and surface-enhanced Raman scattering (SERS) techniques. Experiments performed with R6G (rhodamine 6G) and CV (crystal violet) as Raman reporters demonstrated that the Au@Ag nanostructure amplified SERS signals obviously. Subsequently, the Au@Ag NPs were decorated with 4-mercaptophenylboronic acid (4-MPBA) to specifically recognize glucose by esterification, and a detection limit as low as 10^−4^ M was achieved. Notably, an enhanced linearity for the quantitative detection of glucose (*R*^2^ = 0.995) was obtained after the normalization of the spectral peaks using 4-PATP as the internal standard. Finally, the practical applicability of the developed sensing platform was demonstrated by the detection of glucose in urine with acceptable specificity.

## 1. Introduction

The past decade has witnessed a global spread of diabetes, a serious health problem demanding urgent attention. According to the World Health Organization, diabetes affects over 347 million people worldwide and causes over 3.8 million deaths annually [1,2,3,4,5]. Diabetes results from insulin deficiency, which can increase the levels of glucose in blood [6], those who are affected by this disease have fasting blood glucose concentrations exceeding the physiological values of 3.9–6.2 mM or postprandial blood glucose levels of 3.9–7.8 mM [7], which signifies the importance of blood glucose detection for diagnostics. Among the numerous methods (e.g., fluorescence [8,9], colorimetric [4], near-infrared [10], and electrochemical [2,11,12,13,14]) developed for measuring blood glucose concentrations, electrochemical sensing is viewed as one of the most successful techniques, which relies on the use of glucose oxidase or glucose oxidase/glucose dehydrogenase to generate hydrogen peroxide [15,16]. However, enzymes are generally unstable and highly environmentally sensitive, additionally featuring the drawbacks of easy biological activity loss and high cost [17]. Furthermore, the pain associated with finger piercing during frequent monitoring increases patient discomfort levels. The concentration of glucose in urine is also an important indicator for many diseases, which makes the development of noninvasive or minimally invasive methods of frequent glucose monitoring in urine [18,19,20] 

In recent years, surface-enhanced Raman scattering (SERS) has attracted growing attention due to its highly sensitive and selective detection of biomolecules even on a single-molecule level [21,22,23]. In particular, the pioneering work of label-free SERS-based detection of glucose is demonstrated by employing a 1-decanethiol monolayer adhered on the silver surface to absorb and directly detect glucose [24,25]. However, the above method suffers from low sensitivity and selectivity, because the enrichment of glucose by 1-decanethiol lacks specificity, and glucose exhibits relatively weak Raman activity [26]. It is noted that detection based on the use of Raman reporters affords much stronger signals and is widely used in glucose assays [27,28,29]. For example, phenylboronic acid has been widely used for the detection of saccharides and other diols based on the formation of cyclic boric anhydrides [30,31]. Meanwhile, it has also been demonstrated to utilize the derivatives of mercaptophenylboronic acid (MPBA) for glucose detection, relying on the observation of characteristic Raman peaks in the biologically silent region of 1800–2200 cm^−1^. In addition, the detection of glucose has recently been executed by using a triosmium carbonyl cluster–boronic acid conjugate [32] and an alkyne-functionalized boronic acid [33] as the mid-IR (Infrared ray) probes. 

**Figure 1:**
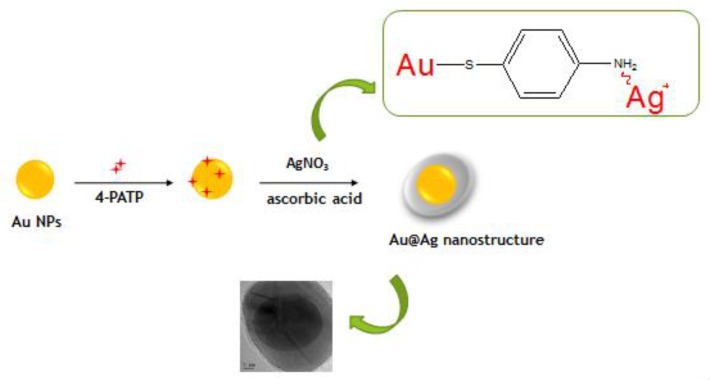
Synthesis of Au@Ag nanostructures containing an internal Raman standard.

Bimetallic nanoparticles (NPs) have received considerable attention in view of their unique optical, magnetic, and other properties that make them potentially well suited for a range of diverse applications. Among these nanostructures, Au@Ag NPs, with strong surface plasmon resonance characteristics, are particularly attractive [34,35,36,37]. Herein, we used 4-aminothiophenol (4-PATP) to connect Au NPs and Ag NPs to form Au@Ag core-shell nanostructures, which are subsequently modified with 4-MPBA to specifically recognize glucose (Figure 1). The use of the small molecules (e.g., 4-PATP) [38] as templates to fabricate intra-gap core-shell structures results in the generation of “hot spots” that can enhance the Raman signal intensity of these molecules [39,40]. Moreover, since 4-PATP molecules presented inside the shell are not subject to the adverse effects of the external environment or desorption, they can also act as the internal standards.

## 2. Materials and Methods 

### 2.1. Materials

Silver nitrate (AgNO_3_), chloroauric acid (HAuCl_4_), and ascorbic acid (C_6_H_8_O_6_) were purchased from Shenbo Chemical Co., Ltd. (Shanghai, China). Trisodium citrate (C_6_H_5_Na_3_O_7_), 4-aminothiophenol (4-PATP, C_6_H_7_NS), 4-mercaptophenylboronic acid (4-MPBA, C_6_H_7_O_2_BS), rhodamine 6G (R6G), and crystal violet (CV) were supplied by Macklin Biochemical Co., Ltd. (Shanghai, China), and D-glucose was purchased from Xilong Scientific Co., Ltd. (Guangdong, China). Human urine samples were provided by Fujian Cancer Hospital. Aluminum sheet (0.1 × 100 mm, 99.0%) were purchased from Sinopharm Chemical Reagent Co., Ltd. All solutions were prepared using distilled water unless otherwise mentioned. All chemicals were used as received without further purification.

### 2.2. Synthesis of Au NPs

Colloidal Au NPs were prepared by the reduction of HAuCl_4_ with sodium citrate. Briefly, a refluxing HAuCl_4_ solution (2.4 × 10^−3^ M, 100 mL) was mixed with trisodium citrate (1 wt%, 1 mL) under magnetic stirring for 15 min. The produced Au NPs were purified by centrifugation-induced precipitation–resuspension (9000 rpm, 20 min, two times) in water. 

### 2.3. Synthesis of Core-Shell NPs 

Three groups of as-prepared Au NPs (2.4 × 10^−4^ M, 20 mL) were mixed with aqueous 4-PATP solutions of three different concentrations (5 × 10^−3^, 5 × 10^−4^, and 5 × 10^−5^ M), respectively, and the resulting mixtures were stirred overnight. Subsequently, non-bonded 4-PATP was removed by two-fold precipitation-resuspension (9000 rpm, 6 min), and the resulting NP suspension was treated with AgNO_3_ (1 mM, 500 µL), L-ascorbic acid (0.1 M, 209 µL), and NaOH (100 mM, 518 µL), and incubated at 60 °C for 2 h. The obtained core-shell NPs were precipitated by centrifugation (9000 rpm, 6 min) and washed twice with water.

### 2.4. Functionalization of NPs by 4-MPBA 

A suspension of as-prepared core-shell NPs (20 mL) was added to an ethanolic solution containing 4-MPBA (1 mM, 20 µL), and the obtained mixture was stirred for 4 h. Non-bound 4-MPBA was removed by centrifugation-induced precipitation-resuspension, and the resulting suspension of functionalized core-shell NPs was stored for future use.

### 2.5. NPs Characterization

#### 2.5.1. Transmission Electron Microscopy (TEM)

A drop of suspension containing NPs was deposited on a carbon-coated copper grid (Electron Microscopy Sciences, Hatfield, PA, USA) and allowed to dry at room temperature. Transmission electron microscopy was performed by using the low-/high-resolution transmission electron microscopy (TEM; JEM-2100, Tokyo, Japan; FEI F20, Columbus, OH, USA) at an accelerating voltage of 200 kV.

#### 2.5.2. UV-Vis Spectrometer

Ultra Violet-vis adsorption spectra were recorded on a UV1902 UV-vis spectrometer (Lengguang Tech., Shanghai, China) at room temperature using a 600 μL black body quartz cuvette with an optical path length of 1 cm.

#### 2.5.3. Raman Measurement

A drop of the reporter-embedded Au@Ag NPs was dispersed thoroughly in distilled water by sonication. A drop of the dispersion was transferred on the aluminum sheet and allowed to dry at room temperature. The aluminum was placed on the stage of a Renishaw in a Via Raman microscope (Renishaw, England) for Raman measurement. Laser intensity at the samples was ~0.34 mW from the 785 nm line of a diode laser for all measurements. The magnification was 20 times. Exposure time for all measurements was 10 s. Each spectrum was the average of 5 scans. Between different Raman sessions, the 520.7 cm^−1^ peak of a silicon wafer was used to calibrate the spectrograph.

#### 2.5.4. EDX Analysis

The element analysis was conducted by EDAX APOLLO (EDAX Inc., Mahwah, NJ, USA). A 2 μL core-shell solution was added to a carbon-coated copper grid (Electron Microscopy Sciences, Hatfield, PA, USA) and allowed to dry at room temperature.

## 3. Results and Discussion

### 3.1. NPs Characterization

**Figure 2:**
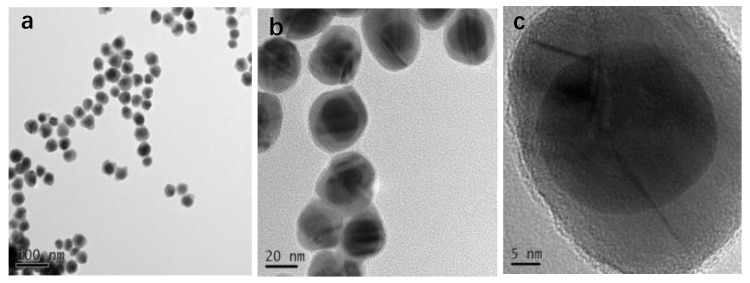

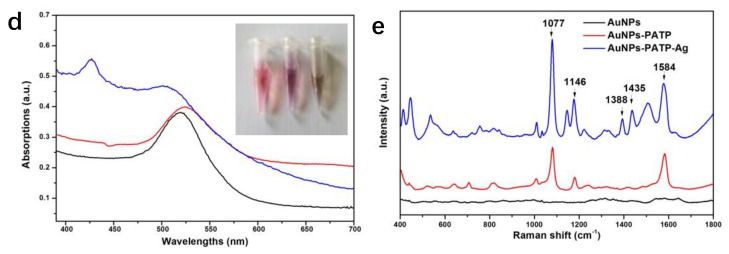
(**a–c**) TEM images of core-shell nanoparticles (NPs) modified with 5 × 10^−4^ M 4-PATP; (**d**) UV-vis absorption spectra of Au NPs, Au-PATP NPs, and Au-PATP-Ag NPs colloids, the inset is the photograph of Au NPs, Au-PATP NPs, and Au-PATP-Ag NPs colloids (from left to right); (**e**) surface-enhanced Raman scattering (SERS) spectra of the metal NPs.

As shown in Figure 1, Au NPs prepared by the reduction of HAuCl_4_ with sodium citrate were treated with aqueous 4-PATP (5 × 10^−4^M). The subsequent introduction of AgNO_3_ (1 mM, 500µL) resulted in the formation of the Au-PATP-Ag^+^ intermediate. After the reduction of Ag+ ions by ascorbic acid, a layer of metallic Ag was produced, forming the Au@Ag core-shell NPs [41]. Figure 2 displays the morphology of as-prepared Au@Ag core-shell NPs. It demonstrates that these particles mostly featured a “poached egg” shape with a dark core (Au) and a light outer shell (Ag). As shown in Figure 2a–c, the obtained NPs had a uniform structure with a 25 nm-thick Au core and a 10 nm-thick Ag shell. The formation of core-shell NPs was accompanied by marked color changes. The treatment of Au NPs with 4-PATP resulted in an immediate color change from red to purple. Subsequently, the solution turned brown green upon the formation of the Ag shell (the inset in Figure 2d). Ultra Violet-vis spectroscopy analysis (Figure 2d) indicated that the plasmon resonance peak of the original Au-NPs at 520 nm shifted to longer wavelengths after the introduction of 4-PATP, which was ascribed to electron transfer between Au NPs and 4-PATP. Upon the formation of the Ag shell, the absorption peak shifted to wavelengths below 520 nm, and a new absorption peak of Ag concomitantly appeared at ~414 nm [35,42]. The structural changes were also monitored by SERS analysis. As shown in Figure 2e, the Au-PATP-Ag core-shell NPs featured enhanced Raman signals of the internal standard, 4-PATP, at 1584, 1435, 1388, 1146 and 1077 cm^−1^ (i.e., all these results indicated that “hot spots” were formed inside the Au@Ag nanostructure) [39,40].

### 3.2. SERS Analysis of NPs Modified with Different Concentrations of 4-PATP 

**Figure 3:**
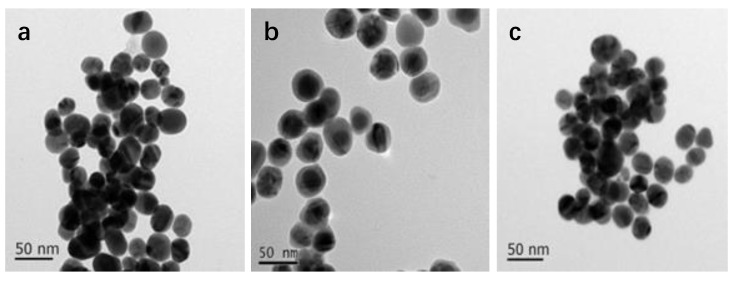

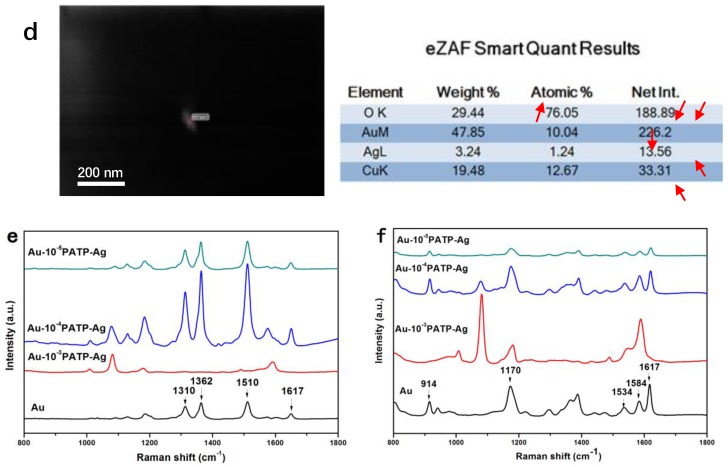
(**a–c**) TEM images of Au@Ag NPs modified with different concentrations of (5 × 10^−5^, 5 × 10^−4^, and 5 × 10^−3^ M, respectively); (**d**) the EDX analysis for Au@Ag NPs modified with 5 × 10^−4^ M with 4-PATP, (**e**,**f**) Raman SERS spectra obtained from R6G and CV with different metal NPs.

The morphology of NPs was tuned by varying the concentration of 4-PATP (5 × 10^−5^, 5 × 10^−4^, and 5 × 10^−3^ M) while keeping the amount of AgNO_3_ constant (1 mM, 500 µL; Figure 3a–c, respectively). Notably, uniform nanostructures were produced only at a 4-PATP concentration of 5 × 10^−4^ M. Results of element analysis revealed that the molar ratio of Au:Ag is 8.10 (Figure 3d), close to the theoretical value of 9.6 (Au: 4.8 μmol; Ag^+^: 0.5 μmol). The quality of as-prepared Au@Ag NPs was probed by employing them to detect R6G (1 × 10^−4^ M) and CV (1 × 10^−4^ M) via SERS (Figure 3e,f). All the spectra, except for the one modified with 5 × 10^−3^ M PATP, clearly exhibit characteristic peaks of R6G molecules at 1310 cm^−1^, 1362 cm^−1^, 1510 cm^−1^, and 1650 cm^−1^, respectively [43]. This rule is also observed in the detection of CV. Except for the red line in Figure 3e, other spectra can well detect the characteristic peaks of CV molecules at 914 cm^−1^, 1170 cm^−1^, 1386 cm^−1^, 1534 cm^−1^, 1584 cm^−1^, and 1617 cm^−1^, respectively [44]. In the case of the metal NPs modified with 5 × 10^−3^ M of PATP, the dense layer of PATP led to the aggregation of Au NPs, thus the hot-spots were formed only for the PATP (sandwiched by Au NPs), this could prevent the acquisition of SERS signals from analyte (i.e., the red line shown in Figure 3e,f are only the signals corresponding to the base). Conversely, a concentration of 5 × 10^−5^ M was too low to enhance the SERS signals of R6G and CV, possibly because PATP-Ag complexation failed to afford a uniform Ag layer on the Au surface [45,46,47]. At the lowest concentration, PAPT was not sufficient to capture the Ag ions to form the core-shell structures, and then the Ag NPs were formed separately (marked by arrows in Figure 3c). Thus, the optimal 4-PATP concentration of 5 × 10^−4^ M was used for the detection of glucose.

### 3.3. Detection of Glucose by SERS

**Figure 4:**
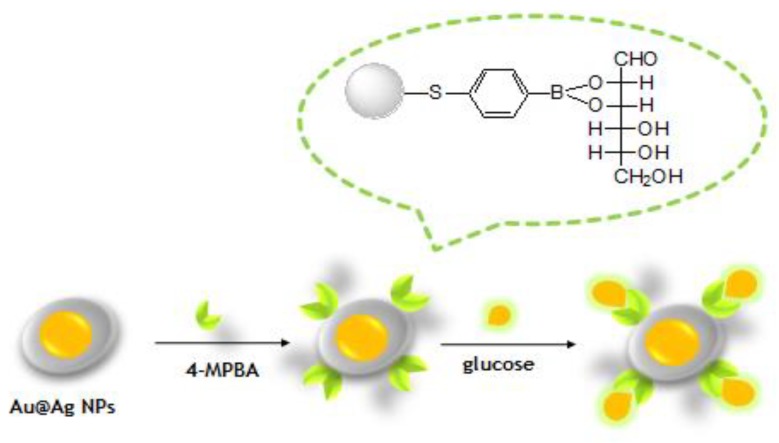
Synthesis of 4-MPBA–modified Au@Ag NPs for glucose detection.

**Figure 5:**
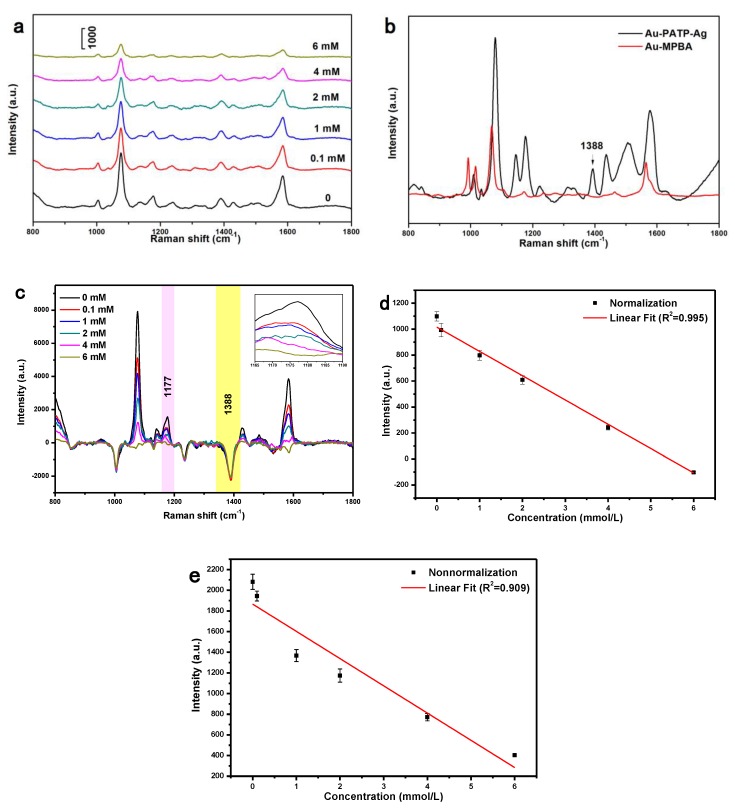
(**a**) Original SERS spectra recorded at different concentrations of glucose; (**b**) SERS spectra of 4-MPBA–modified Au NPs and Au@Ag NPs; (**c**) Normalized SERS spectra recorded at different concentrations of glucose, and the inset is the enlarged view of the peak of 1177 cm^−1^; (**d**–**e**) the working curves of the peak at 1177 cm^−1^ based on normalized SERS spectra and original SERS spectra.

The Au@Ag NPs were then functionalized with 4-MPBA, which enabled the specific recognition of glucose via esterification of the –B(OH)_2_ moiety (Figure 4). Glucose sensing was performed by adding glucose solutions of different concentrations (0.1, 1, 2, 4, and 6 mM) to the colloidal suspensions of as-prepared Au@Ag-MPBA NPs, and the SERS signals of glucose-modified 4-MPBA residues on NPs were recorded after incubation and purification. As shown in Figure 5a, the acquired spectra were dominated by bands at 1077 and 1584 cm^−1^, assigning to in-plane mode–geared βCCC + γCS and γCC modes, respectively., which are often used to identify the adsorption of 4-MPBA on Ag in the deprotonated form to generate Ag–S bonds. Additionally, the intensities of 4-MPBA SERS peaks at 1004, 1077, 1177, and 1584 cm^−1^ decreased with increasing glucose concentration [6,48]. The B–OH stretch at 1177 cm^−1^ was chosen to monitor the binding of glucose with 4-MPBA [49]. Importantly, since the intensity of SERS peaks is influenced by the choice of test points and the surroundings, 4-PATP, commonly regarded as a reporter or aggregator [50,51,52], was herein employed as an internal standard. The band at 1388 cm^−1^ was chosen as the standard peak of 4-PATP, because this peak was observed only for 4-PATP, but not for 4-MPBA [47,49] (Figure 5b). Subsequently, SERS spectra recorded in the presence of different concentrations of glucose were normalized using the above peak as a benchmark (Figure 5c). After normalization, the intensity of the chosen band (Figure 5d) decreased with the increase in concentration of glucose, and better linearity (Figure 5d, *R*^2^ = 0.995) was observed compared to the case when original SERS (Figure 5e, *R*^2^ =0.909) was performed. These observations may be attributed to the fact that the core-shell nanostructures can enhance the SERS signals of the internal standard molecules, and the outer silver shell can protect the internal standard material to be influenced or interfered by the environment.

### 3.4. Detection of Glucose in Urine 

**Figure 6:**
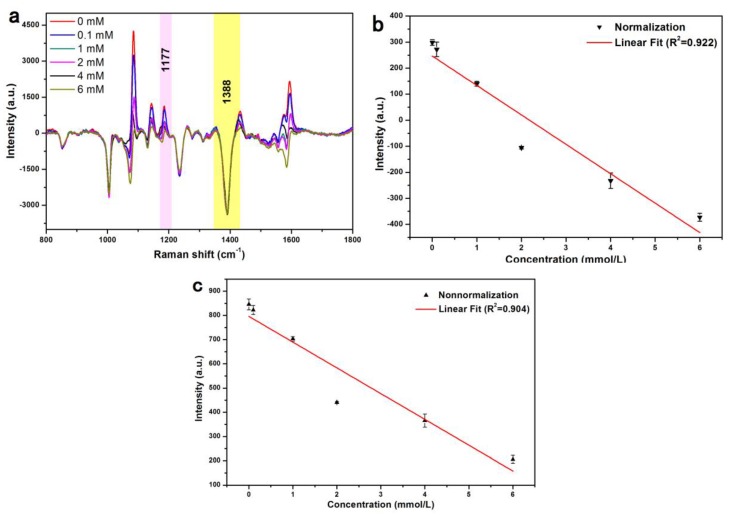
(**a**) Normalized SERS spectra of Au@Ag NPs recorded in the presence of different glucose levels in urine (**b**) the working curve for the peak at 1177 cm^−1^ based on normalized SERS spectra, (**c**) the working curve of the peak at 1177 cm^−1^ based on original SERS spectra.

**Figure 7:**
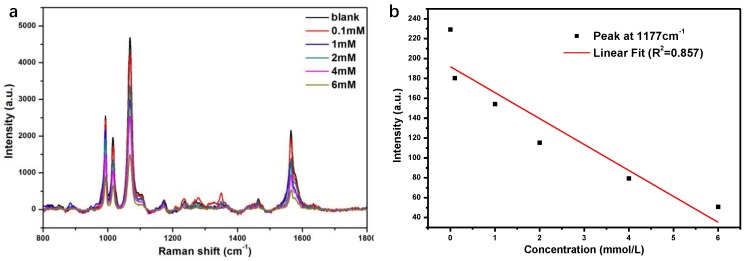
(**a**) The SERS spectra of different concentration glucose detected by using MPBA-modified Ag nanoparticles; (**b**) the linear correlation of Raman peak intensity at 1177 cm^−1^ and the concentration of glucose.

To demonstrate the practical applicability, the developed sensor was employed to trace glucose in undiluted urine samples using the standard addition method (Figure 6a). As in the abovementioned case, the quantitative detection was based on the intensity at a specific wavenumber. The band at 1388 cm^−1^ was chosen as the normalization benchmark, and the response intensity of the 1177 cm^−1^ peak was shown to exhibit better linearity (*R*^2^ = 0.922, Figure 6b) than original spectra (*R*^2^ = 0.904, Figure 6c) despite the presence of potential interferences. In order to strengthen the advantage of Au-Ag core-shell structure, we used the pure Ag modified 4-MPBA to do the same test for comparison. As shown in Figure 7, Ag nanoparticles shows the linearity for the Raman intensity at 1177 cm^−1^-glucose concentration with *R*^2^ = 0.857, this is worse than that of Au-PATP-Ag nanoparticles (*R*^2^ = 0.922, Figure 6b). The enhanced linear correlation could be ascribed to the particular structure of Au-PATP-Ag. The PATP in the particles behaves not only a linker for the formation of Au@Ag nanostructure, but also as the internal substance, which can significantly impair interference from the environment, resulting in the improvement of the veracity in the quantitative test. The linearity in the range of 0.1–6 mM is relatively good, but the linearity is not satisfactory for the concentration below 0.1 mM, so the limit of detection (LOD) in this study is around 0.1mM. The achieved LOD was comparable to those of reported works as listed in Table 1 [4,10,14,18,19,20,32]. Although our LOD do not reach the state of the art, this method is much simpler and cheaper. Thus, these findings of the effective and accurate determination of glucose in urine samples strongly suggested that the developed glucose nanosensor may be suitable for clinical diagnosis applications. 

**Figure 8:**
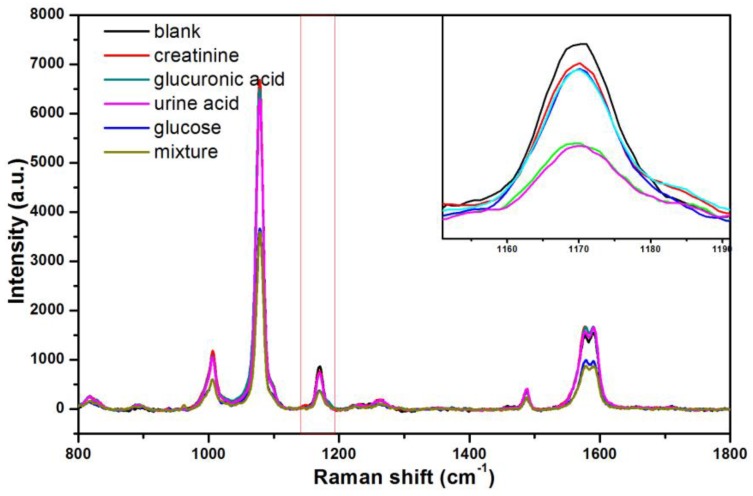
SERS spectrum of glucose, uric acid, glucuronic acid, creatinine solutions, and their mixture with same concentration (10^−3^ M).

To check the selectivity of our method, glucose, uric acid, creatinine, D-glucuronic acid solutions and their mixture with same concentration were prepared for SERS measurement in urine. As shown in Figure 8, the urine samples added with glucuronic acid, uric acid, and creatinine exhibit the SERS signals basically similar to the base signal at characteristic peak of 1177 cm^−1^. However, the glucose can be detected in the urine, and the SERS signal detected in the mixture of four substances is similar to that of the sample with glucose added only. Such observations mean that the method can specifically identify glucose molecules in urine.

## 4. Conclusions

We developed a new method for synthesizing Au@Ag core-shell NPs with internal standards to achieve enhanced, stable, and reproducible SERS signals by employing 4-PATP residing inside the Au@Ag structure as a coordination-prone Raman reporter. The concentration of 4-PATP influenced the structure of as-prepared NPs, and the optimized concentration was determined of 5 × 10^−4^ M. As-prepared Au@Ag NPs were then modified with 4-MPBA and used for quantitative SERS analysis of glucose, with its rapid detection in urine at the concentration as low as 0.1 mM in the presence of other interferents. The strong and specific peaks of the employed internal standard could be used as a benchmark. Moreover, the presence of a shell around 4-PATP reduced the influence of the environment. The described method can potentially be used as a well-defined and easy-to-employ technique for engineering multifunctional SERS-active materials with a wide range of applications for ultrasensitive and quantitative analyte detection.

## Figures and Tables

**Table 1:** Comparison of sensing performance for different detection methods.

Limit of Detection (LOD)	Detection Method	Detection Selectivity	References
0.1 mM	SERS with Au@Ag NPs modified with internal standard	Urine, Creatinine, Uric acid, Glucuronic acid	This work
0.56 mM	Naked eye detection using glucose oxidase	Creatinine, Cysteine	[4]
0.11 mM	Near-infrared (NIR)	Blood	[10]
1 mM	electrochemical method	Uric acid, Ascorbic acid	[14]
0.1 mM	SERS with Ag@Fabric NanoZymes	Urine	[18]
1 mM	SERS with Au NPs substrate	Urine	[19]
10 nM	SERS with nanorods assembled substrate	Galactose, Fructose	[20]
5.1 mM	SERS substrate with metal carbonyl probe	Urine	[32]
